# PSEUDOMARKER 2.0: efficient computation of likelihoods using NOMAD

**DOI:** 10.1186/1471-2105-15-47

**Published:** 2014-02-17

**Authors:** Edward Michael Gertz, Tero Hiekkalinna, Sébastien Le Digabel, Charles Audet, Joseph D Terwilliger, Alejandro A Schäffer

**Affiliations:** 1 National Center for Biotechnology Information, NIH, DHHS, Bethesda, MD, USA; 2 Unit of Public Health Genomics, National Institute for Health and Welfare, Helsinki, Finland; 3 Institute for Molecular Medicine Finland, University of Helsinki, Helsinki, Finland; 4 GERAD and Département de Mathématiques et de Génie Industriel, École Polytechnique de Montréal, Montréal, Canada; 5 Department of Psychiatry, Department of Genetics and Development, and Columbia Genome Center, Columbia University, New York NY, USA; 6 Division of Medical Genetics, New York State Psychiatric Institute, New York NY, USA

## Abstract

**Background:**

PSEUDOMARKER is a software package that performs joint linkage and linkage disequilibrium analysis between a marker and a putative disease locus. A key feature of PSEUDOMARKER is that it can combine case-controls and pedigrees of varying structure into a single unified analysis. Thus it maximizes the full likelihood of the data over marker allele frequencies or conditional allele frequencies on disease and recombination fraction.

**Results:**

The new version 2.0 uses the software package NOMAD to maximize likelihoods, resulting in generally comparable or better optima with many fewer evaluations of the likelihood functions.

**Conclusions:**

After being modified substantially to use modern optimization methods, PSEUDOMARKER version 2.0 is more robust and substantially faster than version 1.0. NOMAD may be useful in other bioinformatics problems where complex likelihood functions are optimized.

## Background

PSEUDOMARKER [1] is a package that genomically localizes trait-predisposing loci by performing statistical tests using a putative disease locus and a series of markers. Genomic localization of genes that impact some phenotype is based on tests of independence of disease phenotypes from genotypes of a genome-spanning set of markers. Many “association tests” try to test directly for statistical relationships between disease phenotypes and marker genotypes directly by sampling large numbers of cases and controls or very small families. Such tests confound the statistical relationship between marker alleles and the genotypes at a putative nearby disease locus with the statistical relationship between the same markers and the phenotype. This confounding is unavoidable for case-control data because of the limited degrees of freedom, but these relationships can and should be modeled explicitly when analyzing more complex and heterogeneous pedigree sets.

PSEUDOMARKER performs a full likelihood analysis under a specified model of the relationship between disease phenotypes and underlying genotypes. In pedigree data, one can test for *genetic linkage* as the preferential cosegregation of a marker or a haplotype with disease family-by-family; the marker genotype that cosegregates with the disease can differ from family to family. In either pedigree data or in case-control data, one can test for *linkage disequilibrium* (LD) between a marker and a putative disease locus as the preferential co-occurrence of a specific genotype at the marker with a genotype at the disease locus. By using a full likelihood model, PSEUDOMARKER can combine analysis of case-control (singletons) data and pedigree data of arbitrary size in one unified testing framework. We directly analyze linkage and LD among marker and disease genotypes, integrating over all possible genotypes at the putative two-allele disease-predisposing locus, for all individuals under an explicit model of the genotype-phenotype relationship.

PSEUDOMARKER version 1 maximizes several likelihood functions [1] using a generalized pattern search (GPS) algorithm [2] implemented in a custom version of the ILINK [3] program. Previously, we showed that PSEUDOMARKER, using GPS likelihood estimates, performed well in detecting linkage and LD, outperforming several competing genetic analysis programs as measured by the power or false positive rate [4].

The running time of PSEUDOMARKER depends on the number of times the optimization algorithm evaluates any likelihood function. Each evaluation involves computation over one or, often, several pedigrees for fixed values of certain parameters that may include the recombination fraction and marker allele frequencies. ILINK computes these likelihoods using a peeling method that is a generalization of the Elston-Stewart algorithm [5]. Computation time is highly dependent on the pedigree structure and the number of untyped founders.

A reduction in the number of likelihood function evaluations would allow more samples, larger and more complex pedigrees, or a greater density of markers to be analyzed in a reasonable amount of time. Although the GPS method [2] was more robust than the older line search method implemented in all previous versions of ILINK, we decided that the number of likelihood evaluations might be reduced by using instead a newer algorithm known to outperform GPS in some other optimization problems.

Mesh Adaptive Direct Search (MADS) [6] is a framework for a class of derivative-free algorithms designed to supersede the GPS method. MADS is conceptually similar to GPS, but uses a richer set of search directions, resulting in better theoretical convergence properties. The NOMAD software package [7] is a high-quality, C++ open-source implementation of MADS algorithms in use in universities and companies around the world [8-11]. NOMAD is robust [12] and has a wide range of functionality, including handling of general nonlinear constraints, biobjective optimization, parallelism, and the restriction of variables to integer or boolean values [13].

We describe PSEUDOMARKER 2.0, which uses a customized version of ILINK that uses NOMAD to maximize likelihoods. We show that NOMAD is more effective at finding optima than GPS, while requiring fewer evaluations of the likelihood function.

## Implementation

### PSEUDOMARKER

PSEUDOMARKER uses parametric inheritance models and exact likelihood computations to evaluate the evidence for linkage and/or LD between a putative trait locus and a set of genotyped markers. When applying extreme parametric models, it yields statistics that are stochastically equivalent to several popular model-free methods if applied to simple family structures [14], for instance mother-father-child triads, case-control samples, or affected sib-pairs. PSEUDOMARKER, however, has substantial advantages over the simpler nonparametric methods when analyzing more complex family structures [1,4].

PSEUDOMARKER takes as input a pedigree file (including pedigree structures and genotypes) in LINKAGE format [15], a common format used by many analysis packages, such as the well-known PLINK package [16]. The map file that describes the names and positions of the markers may be supplied using any one of a variety of formats, including the format used by PLINK. The format of the map file must be explicitly specified as a command-line option. Many more details on the PSEUDOMARKER data input format can be found in the online documentation (http://www.helsinki.fi/~tsjuntun/pseudomarker/, under Tutorial).

PSEUDOMARKER uses likelihood ratio tests to compare four models describing all possible combinations of having or not having linkage and having or not having LD. Marker allele frequencies are parameters of all four likelihood functions. For likelihoods allowing for LD, the marker allele frequencies are allowed to vary conditional on which trait-locus allele is on the same haplotype. For likelihoods allowing for linkage, the probability with which recombination occurs between trait and marker loci (the recombination fraction) is a parameter. For each likelihood function, all parameters are estimated jointly.

Estimating the parameters is a nonlinear constrained optimization problem. ILINK uses the pedigree structure, genomic data and the inheritance model to compute each likelihood function exactly as a nonlinear function of its free parameters. Marker allele frequencies and conditional allele frequencies are probabilities, and as such are constrained to lie between 0 and 1. Each set of frequencies must also sum to 1. The recombination fraction, if a parameter, is constrained to lie between the 0 and 0.5, because larger values of the recombination fraction are not biologically meaningful; a recombination fraction of 0.5 between two loci indicates that the loci segregate independently.

The main programs of PSEUDOMARKER are primarily intended to be used for fine mapping a linkage region as has been done, for example, in Kyöstilä *et al.*[17], and for testing candidate genes as has been done in Deo *et al.*[18]. PSEUDOMARKER may be used for genome-wide data, but for larger or more complex problems using current (*circa* 2014) technology requires the use of a computational cluster to complete the genome-wide analysis in reasonable time. For most purposes, if the data set contains a large number of families, we instead recommend two-stage analysis approach. In the first stage, a filter based on the haplotype-based haplotype relative risk (HHRR) method [19] and less computationally expensive classical linkage analysis with loose thresholds is used to identify markers likely to benefit from PSEUDOMARKER analysis. The second stage performs full PSEUDOMARKER analysis on these candidate markers. A program *twostage.py* is provided in the PSEUDOMARKER distribution to perform the two-stage analysis. A description of the two-stage method and instructions are available on the PSEUDOMARKER website (under Tutorial/Two Stage Analyses).

### NOMAD

NOMAD [7] implements several variants of the MADS framework for constrained derivative-free optimization. In its usual mode, it searches for an optimum by generating trial points along orthogonal directions starting from the incumbent best solution [20]. The set of directions used in this step is far richer than the set of directions searched by GPS; formally, the set of normalized directions is dense in the unit sphere. The use of such a rich set of search directions ensures stronger theoretical convergence properties, and leads to a more efficient algorithm in practice [6]. The MADS framework is flexible enough to allow the use of heuristics that investigate additional trial points to improve practical convergence. Heuristics available in NOMAD include Variable Neighborhood Search (VNS) metaheuristic [21] and the construction and exploration of quadratic models of the objective function and of the constraints [12]. The VNS metaheuristic was not used in our tests, but quadratic models are used by NOMAD in its default mode, and were used in our tests.

To optimize likelihoods, NOMAD proposes to ILINK values for its free parameters, trial points in the MADS framework. ILINK attempts to evaluate the likelihood function at these trial points. NOMAD explicitly handles bound constraints, and so will not, for instance, suggest a negative probability. The constraints that allele frequencies sum to 1 was handled by another of NOMAD’s features, the extreme barrier approach. For any set of marker allele frequencies, one frequency may be represented implicitly, its value obtained by subtracting the sum of the other frequencies from 1. NOMAD is not aware of the implicit frequencies. For a trial point suggested by NOMAD, it is possible for an implicit frequency to have an infeasible value: a negative value or a value greater than one. In such a circumstance, the extreme barrier takes effect. ILINK informs NOMAD that the trial point is infeasible, and NOMAD ignores the point, effectively treating it as if it had an infinitely bad objective value.

ILINK was modified substantially to use NOMAD instead of GPS. We used NOMAD in its library mode [13]. Using NOMAD in this mode involves setting up internal ILINK data structures prior to invoking NOMAD, providing NOMAD with code (a C++ class) that NOMAD uses as a callback to provide ILINK with trial points, and converting between NOMAD’s representation of the variables and ILINK’s, ultimately invoking an internal ILINK routine named *likelihood*. NOMAD was run in a mode that uses 2*n* orthogonal search directions, where *n* represents the number of optimization variables. NOMAD was stopped when the minimum poll size, a NOMAD parameter, was less than 10^-4^, indicating that for the next set of trial points, the largest change to any parameter to the likelihood functions would be at most 10^-4^.

The interface between PSEUDOMARKER and ILINK was modified to enable better performance, but these changes do not affect the file formats or command-line syntax for PSEUDOMARKER. NOMAD is used by default. Compiled executable files are available from the PSEUDOMARKER web site (see Availability and requirements). These files include and will use the NOMAD solver without requiring any additional downloads or user intervention. In accordance with the LGPL version 3.0, downloaded archives also contain files allowing users to rebuild the necessary executables using a different, possibly modified, but application programming interface (API) compatible, version of NOMAD.

### Computational experiments

Table 1 gives a brief summary of the 14 data sets that we analyzed in this project. Twelve of these data sets were used to compare the overall number of likelihood function evaluations required by PSEUDOMARKER to complete the analyses of specific markers when using GPS to the number of evaluations needed to complete the analyses of the same markers when using NOMAD. Two additional data sets were used to compare processor time used by PSEUDOMARKER version 1.06d (the last release with major version 1) to that used by PSEUDOMARKER 2.0, to complete realistic genomic scans of chromosome 22. Table 2 shows pedigree statistics of the data sets; more detailed statistics are shown in Additional file 1: Tables S1–S3. Pedigree, phenotype, and marker statistics were computed using PedStats [22].

**Table 1:** Summary of all data sets

**Test set**	**Description**	**Reference**
fin1	Familial combined hyperlipidemia pedigrees from Finland	Pajukanta *et al.*[23]
fin2	Migraine pedigrees from Finland	Wessman *et al.*[24], Kaunisto *et al.*[25], Hiekkalinna *et al.*[4]
fin3	A sub set of the Migraine families (different phenotype and genotyped individuals than on data set fin2)	Tikka-Kleemola *et al.*[26]
fin4	Schizophrenia families from Finland	Ekelund *et al.*[27], Hiekkalinna *et al.*[4]
fin5	Same as fin1, but with multiallelic markers	
fin6	Same as fin1, but with highly polymorphic marker	
x.linked	Extended pedigrees and triads from northern Finland with real X-chromosomal marker data	Karjalainen *et al.*[28]
100sibs	Artificial sib-pair pedigrees	Hiekkalinna [29]
100sibs.c	Artificial sib-pair pedigrees with additional cases	Hiekkalinna [29]
100sibs.cc	Artificial sib-pair pedigrees with additional cases and controls	Hiekkalinna [29]
mixed	Various size artificial pedigrees (triads, sib-pairs, and extended pedigrees)	Hiekkalinna [29]
noparents	Artificial affected sib-pairs with no parental genotypes	Hiekkalinna [29]
FHS	Framingham Heart-Study marker data and phenotypes	Larson *et al.*[30]
FinnTwin12	Finnish twins and twin families	Kaprio *et al.*[31,32], Törnwall *et al.*[33]

**Table 2:** Data set properties

**Data set**	**Pedigrees**	**Average**	**Singleton**	**Singleton**	**Number of**	**Maxium**
		**pedigree size**	**cases**	**controls**	**markers**	**alleles/marker**
fin1	61	15.33	200	200	3	2
fin2	84	13.08	200	200	3	2
fin3	37	13.24	100	100	4	4
fin4	438	5.79	0	199	3	2
fin5	61	15.33	200	200	4	8
fin6	61	15.33	200	200	1	18
x.linked	482	3.17	112	203	1	20
100sibs	100	4.00	0	0	1	3
100sibs.c	100	4.00	200	0	1	3
100sibs.cc	100	4.00	200	200	1	3
mixed	180	5.22	0	50	6	3
noparents	200	4.50	100	100	2	4
FHS	216	27.68	0	0	2181	2
FinnTwin12	171	3.46	0	0	8502	2

The 12 test data sets used to compare iteration counts were selected to include difficult cases, including such factors as real life pedigree structures, realistic amounts of missing data, and large multi-generational families. Data sets contained both biallelic markers and multiallelic microsatellites. The real data sets were from Finnish gene mapping studies on which TH and JDT were collaborators [24,27], while the simulated data sets were generated as part of the Ph.D. dissertation of TH, some of which have been analyzed in prior publications [1,29]. Simulated genotype data were generated using a modified version of SLINK [34,35]; parameters used for the simulations are shown in Additional file 2: Tables S4 and S5.

Some data sets were observed to present difficult maximization problems for the GPS while the previous version of the PSEUDOMARKER package was being developed. The x.linked test set [28] was particularly interesting because it was x-linked, had multiple alleles, and most of the data were triads, and still maximization was quite time-consuming.

All 12 sets used to compare iteration counts were analyzed under assumptions of both the dominant and recessive extreme inheritance models described in [14] and all four likelihood functions used by PSEUDOMARKER, testing for linkage and/or LD. Six were also analyzed under more biologically plausible inheritance models. We optimized likelihoods using either GPS as previously described [1] or NOMAD [7].

To test running time on real data, we used two data sets: a subset of data from the Framingham Heart Study (FHS) [30] version 18 as deposited in NCBI’s dbGaP, and subset of data from the FinnTwin12 study [31,32]. The FinnTwin12 data were recently used in a joint analysis of linkage and LD [33]. Usage of the FHS data for this purpose is covered by an IRB-approved protocol (Ivan Ovcharenko, Principal Investigator; AAS, Associate Investigator). The FinnTwin12 study was approved by the Ethics Committee of Helsinki University Hospital District and individuals in the study gave their written informed consent.

For the FHS study, phenotypes for heart disease were used only from individuals who had consented to have their data used for general research usage (GRU). Using in-house programs, we extracted data on pedigrees each of which includes at least two individuals who were phenotyped for heart disease. Data were filtered with PLINK [16] to remove most inconsistent markers and to keep only markers such that *r*^2^<0.6 pairwise. A few inconsistent markers that were not detected by PLINK, were detected by PedCheck [36] and also removed. The removal of inconsistent markers is needed here to do comparisons with PSEUDOMARKER version 1. One of several user-interface improvements in PSEUDOMARKER version 2 is the implementation of a command line option –skipmendelerrors to skip over markers with inconsistent genotypes. Pedigree and marker statistics for the filtered pedigree and marker data are shown in Table 2 and Additional file 1: Table S2. Markers were divided into 44 groups of approximately 50 markers and all groups were processed in parallel separately using PSEUDOMARKER version 1 and using PSEUDOMARKER version 2 on a cluster of Linux machines.

The FinnTwin2 data contained phenotype and genotype information from 226 individuals in 171 pedigrees (sibships and triads). Some individuals in the study were twins; for monozygotic twins, only one twin was genotyped in the data analyzed [33]. Pedigree statistics for the filtered pedigree and marker data are shown in Table 2 and Additional file 1: Table S2. Data for all 8502 markers were analyzed in a single run of either PSEUDOMARKER version 1 or PSEUDOMARKER version 2 on a Linux machine. Each analysis was repeated ten times and reported running time is the mean of the time for ten analyses.

## Results and discussion

The numbers of likelihood function evaluations for each test set, summed over all markers, all models, and all maximized likelihood functions, are shown in Table 3. NOMAD is superior in terms of function evaluations to GPS on all test sets. As we discuss below, NOMAD is invoked somewhat differently from GPS on the same optimization problems, which contributes to the improvement.

**Table 3:** Number of function evaluations used by GPS and NOMAD

**Test set**	**GPS**	**NOMAD**	**Test set**	**GPS**	**NOMAD**
fin1	7,650	3,342	100sibs	10,003	3,933
fin2	7,430	3,341	100sibs.c	10,891	3,240
fin3	81,887	10,765	100sibs.cc	7,137	2,811
fin4	8,460	3,250	mixed	39,522	12,278
fin5	83,272	32,662	noparents	34,590	9,143
fin6	284,069	96,626	x.linked	470,517	140,986

We chose as our figure of merit the number of likelihood evaluations because that separates the likelihood evaluation of each PSEUDOMARKER hypothesis and gives an “apples-to-apples” comparison of GPS and NOMAD. Nevertheless, the figure of merit that matters more to users of PSEUDOMARKER is the running time for combined evaluation of all hypotheses. The reduction in number of likelihood evaluations does convert in a linear manner to reduction in running time, but the constants depend on the problem instance. For example, on the full PSEUDOMARKER run of the FHS problem, running time decreased from 88 hours and 24 minutes to 21 hours and 45 minutes, a 4.1-fold reduction. For FinnTwin12, the improvement in running time was even more substantial, decreasing from 38 hours and 5 minutes to 6 hours and 8 minutes, a 6.2-fold reduction.

In preliminary tests, we observed NOMAD was more robust than GPS in finding an optimum (data not shown). There were no obvious patterns to distinguish the problem instances on which NOMAD found a better likelihood value than did GPS. Because NOMAD was more robust, we experimented with invoking NOMAD less often. For GPS, it was often helpful to retry a given optimization problem, using the solution previously returned from GPS as the new starting point because that would sometimes lead to the identification of a better likelihood value. The purpose of these restarts is to encourage convergence to a global optimum, and to reduce the probability that GPS would stall at a non-optimal point. The restarts were unnecessary with NOMAD. Nor was it helpful to start NOMAD at several different initial estimates, as was done with GPS. The counts in Table 3 are counts for invoking NOMAD once to solve each optimization problem, whereas GPS was invoked as described in [1].

Despite the fewer calls to the optimization algorithm, the optimum returned by NOMAD was usually better than the one from GPS. Of the 288 optimization problems we tried based on the first 12 test sets, NOMAD found an assignment to the variables that yielded a log likelihood that was at least 0.005 worse than the value reported by GPS only seven times (see Table 4 and Additional file 3: Table S6). In contrast, NOMAD reported 68 objective values better by at least 0.005 than the values reported by GPS. We considered differences less than 0.005 in the log likelihood to be insubstantial, as such differences would change log of the likelihood ratio by at most 0.01. NOMAD returned answers with objective value more than 0.5 better than GPS 21 times, with the largest difference being 28, a shockingly large value. In contrast, the most GPS improved the objective value over NOMAD was 0.1.

**Table 4:** Changes in objective function

**Data set**	***≤-0.5***	***≤-0.05***	***≤-0.005***	***≥0.005***	***≥0.05***	***≥0.5***
fin3	0	0	0	23	15	8
fin4	0	1	1	0	0	0
fin5	0	0	0	20	13	7
fin6	0	0	0	8	4	2
x.linked	0	2	6	4	4	1
mixed	0	0	0	2	2	2
noparents	0	0	0	11	7	1

Count of changes in the objective function more extreme than the indicated number. Positive changes indicate that NOMAD found the better objective value.

Among the 55 cases for which the objective value changed by at least 0.005 in the numerator in the likelihood ratio test, there were two in which the p-value for the test against hypothesis H0 improved by at least one order of magnitude (Additional file 3: Table S6). In the majority of cases, both programs find similar p-values, though GPS requires more iterations and computer time. Since tests are based on likelihood ratios, which code attains a lower p-value depends on whether a better maximum is found for the numerator or denominator of the likelihood ratio. However, when the p-values differ, p-values produced by NOMAD better supported by the underlying statistical model, whereas p-values produced by GPS-based code represent a failure to maximize the likelihoods.

In [1], we reported that one of the difficulties in GPS is the sum constraint that the allele frequencies have to sum to 1.0. The editor suggested that an alternative method to handle the sum constraints is the generalized logit transformation, which has been shown to work in some other settings [37]. In the PSEUDOMARKER application, we believe that the generalized logit would perform poorly because the maximum likelihood estimate of some probabilities is precisely zero, and this is a frequent occurrence. Under the logit transformation, NOMAD would be tasked with finding a minimizer that had a finite objective value, but for which one of the variables was negatively infinite. This case breaks the assumptions of the theoretical convergence theory of NOMAD [6], and poses practical problems for the implementation.

NOMAD is a constrained code, and is designed to handle bounds on the variables, so working in probability space poses no great problem to it, and we believe this is one of the reasons NOMAD performs better than GPS in the PSEUDOMARKER setting.

## Conclusions

The new PSEUDOMARKER 2.0 has been released (see Availability and requirements) and it uses NOMAD [7] to maximize likelihoods. The new version usually provides better or comparable answers, while using far fewer evaluations of the likelihood functions. Several of the most prominent developers of pedigree analysis methods recognized decades ago that the optimization problems that arise in genetic analysis of pedigrees can be difficult to solve and can benefit from new methods [38-40]. We have shown in this study that MADS methods are more effective than previous methods on the optimization problems that arise in usage of PSEUDOMARKER. Therefore, our work is novel in two major respects. First, in the context of PSEUDOMARKER and pedigree likelihood optimization, the shorter analysis time and increased robustness allow analysis to be attempted on larger data sets and more complex family structures. Second, we introduce a generally useful optimization package, NOMAD, to the bioinformatics and genetic epidemiology communities, where NOMAD may find additional usages.

## Availability and requirements

**Project name:** PSEUDOMARKER 2.0

**Project home page:**http://www.helsinki.fi/~tsjuntun/pseudomarker/

**Operating system(s):** GNU/Linux Intel 64-bit architecture

**Programming language:** C and C++

**Other requirements:** none

**License:** PSEUDOMARKER is a binary distribution with registration required. (PSEUDOMARKER from this site without registration.) NOMAD is distributed with PSEUDOMARKER under terms of the LGPL 3.0.

**Any restrictions to use by non-academics:** no

## Abbreviations

LD: linkage disequilibrium; GPS: generalized pattern search; MADS: mesh adaptive direct search; FHS: Framingham Heart Study.

## Competing interests

The authors declare that they have no competing interests.

## Authors’ contributions

EMG modified FASTLINK to use NOMAD, performed all the experiments, except the test of TwinFinn12, wrote the first draft of the manuscript, and coordinated the manuscript revision. EMG and TH updated PSEUDOMARKER to use NOMAD. TH collected test sets, wrote test scripts, performed the test of TwinFinn12, updated the software documentation, and edited the manuscript. CA and SLD helped integrate NOMAD with FASTLINK, suggested tests, and edited the manuscript. JDT suggested tests and edited the manuscript. AAS conceived the project, helped integrate NOMAD with FASTLINK, suggested tests, prepared the FHS data from dbGaP, and edited the manuscript. All authors read and approved the final manuscript.

## Supplementary Material

Additional file 1 **Tables S1–S3.** Showing statistical information about the test sets. Click here for file

Additional file 2 **Tables S4–S5.** Showing parameters used to generate the simulated genotypes in the test sets. Click here for file

Additional file 3 **Tables S6.** Showing differences in the objective value computed by GPS and by NOMAD. Click here for file
