# Supplementary material for: PSEUDOMARKER 2.0: efficient computation of likelihoods using NOMAD
Source: BMC Bioinformatics. 2014 Feb 17;15:47. doi: 10.1186/1471-2105-15-47 (PMC3932042; doi:10.1186/1471-2105-15-47)
Supplement: Additional file 1 — Tables S1–S3. Showing statistical information about the test sets. [file 1471-2105-15-47-S1.pdf]

Table S1. Pedigree statistics for the real-life data sets fin1, fin2, fin3, fin4, x.linked, FHS, and FinnTwin12.

		fin1	fin2	fin3	fin4	x.linked	FHS	FinnTwin12
**Pedigrees**		61	84	37	438	482	216	171[b]
**Individuals**		935	1099	490	2535	1529	5979	591
**Founders**		319	366	178	914	982	1935	342
**Average pedigree size**		15.33 (3-51)	13.08 (4-47)	13.24 (6-34)	5.79 (3-14)	3.17 (3-33)	27.68 (3-639)	3.46 (3-4)
**Generations (%)**	**2**	14.8	11.9	-	99.5	98.5	11.1	100
	**3**	70.5	56.0	59.5	0.5	0.8	51.9	-
	**4**	13.3	32.1	37.8	-	0.4	31.5	-
	**5**	1.4	-	2.7	-	-	5.5	-
**Phenotyped**	**All**	231 (24.7%)	398 (36.2%)	184 (37.6%)	918 (36.2%)	1307 (85.5%)	1036 (17.3%)	226(38.2%)
	**Founders**	2 (0.6%)	26 (7.1%)	21 (11.8%)	60 (6.6%)	932 (94.9%)	219 (11.3%)	0
**Genotyped**	**All**	676 (72.3%)	810 (73.3%)	351 (71.6%)	1906 (75.2%)	1112 (72.7%)	45-52%[a]	37-41%[a]
	**Founders**	133 (41.7%)	147 (40.2%)	60 (33.7%)	442 (48.4%)	792 (80.7%)	28.1%	0
**Additional singletons**	**Cases**	200	200	100	-	112	0	0
	**Controls**	200	200	100	199	203	0	0

[a]The proportion of genotyped individuals varied over a set of markers on chromosome 22.
[b] Pedigrees consisted of sibships and triads of ungenotyped founders with genotyped children.

Table S2. Pedigree statistics for the artificial data sets 100sibs, 100sibs.c, 100sibs.cc, mixed, and noparents.

		100sibs	100sibs.c	100sibs.cc	mixed	noparents
**Pedigrees**		100	100	100	180	200
**Individuals**		400	400	400	940	900
**Founders**		200	200	200	420	400
**Average pedigree size**		4	4	4	5.22 (3 to 16)	4.50 (4 to 5)
**Generations (%)**	**2**	100	100	100	83.3	100
	**3**	-	-	-	16.7	-
**Phenotyped**	**All**	400 (100%)	400 (100%)	400 (100%)	940 (100%)	900 (100%)
	**Founders**	400 (100%)	400 (100%)	400 (100%)	420 (100%)	400 (100%)
**Genotyped**	**All**	400 (100%)	400 (100%)	400 (100%)	940 (100%)	500 (55.6%)
	**Founders**	400 (100%)	400 (100%)	400 (100%)	420 (100%)	0 (0%)
**Additional singletons**	**Cases**	-	200	200	-	100
	**Controls**	-	-	200	50	100

Table S3. Markers and number of alleles per marker in each data set.

Test set	Marker names	Number of alleles
fin1	SNP[*]	2
fin2	SNP[*]	2
fin3	STR[1,2,3,4]	4
fin4	SNP[*]	2
fin5	STR[1,2]	4
fin5	STR3	5
fin5	STR4	8
fin6	STR1	18
100sibs	STR10	3
100sibs.c	STR10	3
100sibs.cc	STR10	3
mixed	SNP[1,2,3]	2
mixed	STR[1,2,3]	3
noparents	STR[3,4]	4
x.linked	X-STR	20
FHS	rs[**]	2
FinnTwin12	rs[**]	2

*) Each data set had three SNP markers.

**) FHS had 2181 SNP markers; FinnTwin12 has 8502 SNP markers.
