# Supplementary material for: PSEUDOMARKER 2.0: efficient computation of likelihoods using NOMAD
Source: BMC Bioinformatics. 2014 Feb 17;15:47. doi: 10.1186/1471-2105-15-47 (PMC3932042; doi:10.1186/1471-2105-15-47)
Supplement: Additional file 2 — Tables S4–S5. Showing parameters used to generate the simulated genotypes in the test sets. [file 1471-2105-15-47-S2.pdf]

Table S4. Simulation parameters for genomic data used in test sets (sets not listed used real, not simulated, data).

Test set	Marker	P(D)	P(A|D/D)	P(A|D/+)	P(A|+/+)	θ	P(1|D)	P(2|D)	P(3|D)	P(4|D)	P(5|D)	P(1|+)	P(2|+)	P(3|+)	P(4|+)	P(5|+)	Linkage	LD
fin1	SNP_D01	0.1	0.168	0.168	0.084	0.01	0.95	0.05	-	-	-	0.01	0.99	-	-	-	Y	Y
	SNP_D02	0.1	0.168	0.168	0.084	0.01	0.9	0.1	-	-	-	0.01	0.99	-	-	-	Y	Y
	SNP_D12	0.1	0.168	0.168	0.084	0.01	0.4	0.6	-	-	-	0.07	0.93	-	-	-	Y	Y
fin2	SNP_D01	0.1	0.168	0.168	0.084	0	0.95	0.05	-	-	-	0.01	0.99	-	-	-	Y	Y
	SNP_D08	0.1	0.168	0.168	0.084	0	0.6	0.4	-	-	-	0.04	0.96	-	-	-	Y	Y
	SNP_D07	0.1	0.168	0.168	0.084	0	0.65	0.35	-	-	-	0.039	0.961	-	-	-	Y	Y
fin3	STR1	0.1	*	*	*	0.5	0.2	0.6	0.1	0.1	-	**	**	**	**	-	N	N
	STR2	0.1	0.5	0.5	0.009	0.1	0.1	0.7	0.1	0.1	-	**	**	**	**	-	Y	N
	STR3	0.1	0.5	0.5	0.009	0	0.1	0.7	0.1	0.1	-	**	**	**	**	-	Y	N
	STR4	0.1	0.5	0.5	0.009	0	0.1	0.7	0.1	0.1	-	0.1	0.4	0.4	0.1	-	Y	Y
fin4	SNP_D07	0.1	0.06	0.009	0.009	0	0.8	0.2	-	-	-	0.01	0.99	-	-	-	Y	Y
	SNP_D17	0.1	0.06	0.009	0.009	0	0.15	0.85	-	-	-	0.0875	0.9125	-	-	-	Y	Y
	SNP_D04	0.1	0.06	0.009	0.009	0	0.65	0.35	-	-	-	0.01	0.99	-	-	-	Y	Y
fin5	STR1	0.1	0.5	0.5	0.05	0	0.4	0.01	0.01	0.58	-	0.1	0.01	0.01	0.88	-	Y	Y
	STR2	0.1	*	*	*	0.5	0.94	0.03	0.02	0.1	-	**	**	**	**	-	N	N
	STR3	0.1	0.5	0.5	0.05	0	0.35	0.02	0.03	0.3	0.3	0.15	0.02	0.03	0.5	0.3	Y	Y
	STR4	0.1	*	*	*	0.5	(See allele frequencies in Table S6)										N	N
fin6	STR1	0.1	0.168	0.168	0.084	0	(See allele frequencies in Table S6)										Y	Y
100sibs[.c, .cc]	STR10	0.1	0.5	0.5	0.05	0.01	0.2	0.6	0.2	-	-	0.2	0.2	0.6	-	-	Y	Y
mixed	SNP1	0.01	*	*	*	0.5	0.5	0.5	-	-	-	**	**	-	-	-	N	N
	SNP2	0.01	0.9	0.9	0.01	0.1	0.5	0.5	-	-	-	0.5	0.5	-	-	-	Y	N
	SNP3	0.01	0.9	0.9	0.01	0.1	0.7	0.3	-	-	-		0.7	-	-	-	Y	Y
	STR1	0.01	*	*	*	0.5	0.33	0.33	0.34	-	-	**	**	**	-	-	N	N
	STR2	0.01	0.9	0.9	0.01	0.1	0.33	0.33	0.34	-	-	0.33	0.33	0.34	-	-	Y	N
	STR3	0.01	0.9	0.9	0.01	0.1	0.7	0.2	0.1	-	-	0.1	0.2	0.7	-	-	Y	Y
noparents	STR3	0.1	0.5	0.05	0.05	0.01	0.25	0.25	0.25	0.25	-	0.25	0.25	0.25	0.25	-	Y	N
	STR4	0.1	0.5	0.05	0.05	0.01	0.7	0.1	0.1	0.1	-	0.1	0.1	0.1	0.7	-	Y	Y

*) Inheritance model is irrelevant when there is no linkage and no linkage disequilibrium (LD); **) when there is no LD, then P(1|D)=P(1|+), P(2|D)=P(2|+), etc.

Table S5. Marker allele frequences for two simulated microsatellites

Test set	Marker	P(1)	P(2)	P(3)	P(4)	P(5)	P(6)	P(7)	P(8)
fin5	STR4	0.1	0.02	0.2	0.15	0.001	0.002	0.002	0.525

Test set	Marker	P(1|D)	P(2|D)	P(3|D)	P(4|D)	P(5|D)	P(6|D)	P(7|D)	P(8|D)	P(9|D)	P(10|D)	P(11|D)	P(12|D)	P(13|D)	P(14|D)	P(15|D)	P(16|D)	P(17|D)	P(18|D)	P(19|D)	P(20|D)
fin6	STR1[§]	0.06	0.002	0.03	0.02	0.000002	0.002	0.02	0.02	0.18	0.002	0.002	0.2	0.002	0.1	0.002	0.3	0.002	0.002	0.0374	0.016598
		P(1|+)	P(2|+)	P(3|+)	P(4|+)	P(5|+)	P(6|+)	P(7|+)	P(8|+)	P(9|+)	P(10|+)	P(11|+)	P(12|+)	P(13|+)	P(14|+)	P(15|+)	P(16|+)	P(17|+)	P(18|+)	P(19|+)	P(20|+)
		0.06	0.002	0.02	0.18	0.2	0.002	0.02	0.02	0.18	0.002	0.002	0.002	0.002	0.1	0.002	0.03	0.002	0.002	0.00002	0.17198

[§]Simulation parameters included allele frequencies for 20 alleles, but only 18 alleles appeared in the simulated genotype data.
