# Supplementary material for: PSEUDOMARKER 2.0: efficient computation of likelihoods using NOMAD
Source: BMC Bioinformatics. 2014 Feb 17;15:47. doi: 10.1186/1471-2105-15-47 (PMC3932042; doi:10.1186/1471-2105-15-47)
Supplement: Additional file 3 — Tables S6. Showing differences in the objective value computed by GPS and by NOMAD. [file 1471-2105-15-47-S3.pdf]

Table S6. Improvement in optimal log likelihood when using NOMAD to find the optimum, instead of generalized pattern search (GPS). Positive numbers indicate that NOMAD found the better objective value; negative numbers indicate that GPS was better. Differences of magnitude less than 0.005 are not reported, so 75 differences are reported out of 288 total instances. See Hiekkalinna *et al*. (2011) for an explanation of the hypotheses H0-H3. Reported p-values are for testing the indicated hypothesis against H0. H0 is never tested against itself, so p-values are reported as not applicable (NA) for those rows. **Bold** rows indicate that the optimum obtained by NOMAD was at least 0.5 better than the optimum obtained by the GPS.

Test Set	Inheritance	Hypothesis	Marker	Improvement in log likelihood	P-value vs. H0	
					GPS	NOMAD
**mixed**	**Model-based**	**H2**	**SNP2**	**1.209**	**0.26**	**0.12**
**mixed**	**Model-based**	**H3**	**SNP2**	**0.810**	**7.4e-07**	**4.9e-07**
noparents	Dominant	H0	STR4	0.005	NA	NA
**noparents**	**Dominant**	**H2**	**STR4**	**5.241**	**0.42**	**0.044**
noparents	Dominant	H3	STR4	0.332	0.00018	0.00016
noparents	Recessive	H0	STR4	0.005	NA	NA
noparents	Recessive	H2	STR4	0.087	0.18	0.18
noparents	Recessive	H3	STR4	0.109	2.2e-05	2.1e-05
noparents	Model-based	H0	STR4	0.005	NA	NA
noparents	Model-based	H1	STR4	0.009	1.7e-07	1.7e-07
noparents	Model-based	H2	STR4	0.445	0.88	0.77
noparents	Model-based	H3	STR4	0.114	4e-06	4e-06
noparents	Model-based	H2	STR5	0.083	8.5e-14	8.2e-14
x-linked	Dominant	H0	X-STR	0.199	NA	NA
x-linked	Dominant	H2	X-STR	-0.020	0.1	0.11
x-linked	Dominant	H3	X-STR	-0.041	0.0089	0.0095
x-linked	Recessive	H0	X-STR	0.199	NA	NA
**x-linked**	**Recessive**	**H1**	**X-STR**	**0.507**	**0.5**	**0.48**
x-linked	Recessive	H2	X-STR	-0.042	0.0083	0.0089
x-linked	Recessive	H3	X-STR	-0.102	0.0031	0.0034
x-linked	Model-based	H0	X-STR	0.199	NA	NA
x-linked	Model-based	H2	X-STR	-0.036	0.1	0.11
x-linked	Model-based	H3	X-STR	-0.053	0.012	0.013
fin3	Dominant	H2	STR1	0.013	0.13	0.13
fin3	Dominant	H0	STR2	0.103	NA	NA
fin3	Dominant	H1	STR2	0.138	0.027	0.026
fin3	Dominant	H2	STR2	0.375	0.94	0.88
**fin3**	**Dominant**	**H3**	**STR2**	**0.705**	**0.27**	**0.21**
fin3	Dominant	H0	STR3	0.281	NA	NA
fin3	Dominant	H1	STR3	0.090	0.00015	0.00016
**fin3**	**Dominant**	**H2**	**STR3**	**1.602**	**0.63**	**0.39**
**fin3**	**Dominant**	**H3**	**STR3**	**1.009**	**0.0059**	**0.0042**
**fin3**	**Dominant**	**H0**	**STR4**	**2.315**	**NA**	**NA**
**fin3**	**Dominant**	**H2**	**STR4**	**14.502**	**0.12**	**0.00042**
**fin3**	**Dominant**	**H3**	**STR4**	**28.198**	**3.5e-07**	**1.4e-12**
fin3	Recessive	H3	STR1	0.028	0.011	0.011
fin3	Recessive	H0	STR2	0.103	NA	NA

fin3	Recessive	H1	STR2	0.115	0.016	0.016
fin3	Recessive	H2	STR2	0.235	0.98	0.95
fin3	Recessive	H3	STR2	0.050	0.24	0.25
fin3	Recessive	H0	STR3	0.281	NA	NA
fin3	Recessive	H1	STR3	0.064	0.00089	0.001
fin3	Recessive	H2	STR3	0.431	0.89	0.85
fin3	Recessive	H3	STR3	0.383	0.029	0.027
**fin3**	**Recessive**	**H0**	**STR4**	**2.315**	**NA**	**NA**
**fin3**	**Recessive**	**H2**	**STR4**	**4.489**	**0.031**	**0.011**
fin4	Dominant	H3	SNP_D17	-0.057	0.51	0.53
fin5	Dominant	H0	STR1	0.033	NA	NA
**fin5**	**Dominant**	**H0**	**STR2**	**0.733**	**NA**	**NA**
fin5	Dominant	H1	STR2	0.332	0.21	0.31
**fin5**	**Dominant**	**H2**	**STR2**	**0.941**	**0.6**	**0.55**
fin5	Dominant	H3	STR2	0.297	0.42	0.48
**fin5**	**Dominant**	**H2**	**STR3**	**1.916**	**0.001**	**0.00043**
**fin5**	**Dominant**	**H3**	**STR3**	**1.493**	**6.8e-05**	**3.5e-05**
fin5	Dominant	H0	STR4	0.081	NA	NA
fin5	Dominant	H1	STR4	0.085	0.46	0.46
fin5	Dominant	H3	STR4	0.029	0.58	0.58
fin5	Recessive	H0	STR1	0.033	NA	NA
**fin5**	**Recessive**	**H0**	**STR2**	**0.733**	**NA**	**NA**
**fin5**	**Recessive**	**H1**	**STR2**	**0.717**	**0.2**	**0.21**
fin5	Recessive	H2	STR2	0.135	0.77	0.92
fin5	Recessive	H3	STR2	0.482	0.63	0.68
**fin5**	**Recessive**	**H2**	**STR3**	**1.446**	**0.001**	**0.00052**
fin5	Recessive	H0	STR4	0.081	NA	NA
fin5	Recessive	H1	STR4	0.020	0.28	0.3
fin5	Recessive	H2	STR4	0.025	0.37	0.38
fin5	Recessive	H3	STR4	0.019	0.21	0.22
fin6	Dominant	H0	STR1	0.030	NA	NA
fin6	Dominant	H1	STR1	0.039	0.068	0.067
**fin6**	**Dominant**	**H2**	**STR1**	**0.690**	**0.74**	**0.69**
fin6	Dominant	H3	STR1	0.285	0.61	0.6
fin6	Recessive	H0	STR1	0.030	NA	NA
fin6	Recessive	H1	STR1	0.023	0.062	0.062
**fin6**	**Recessive**	**H2**	**STR1**	**0.540**	**0.34**	**0.31**
fin6	Recessive	H3	STR1	0.204	0.24	0.23
